# An engineered interleukin‐11 decoy cytokine inhibits receptor signaling and proliferation in lung adenocarcinoma

**DOI:** 10.1002/btm2.10573

**Published:** 2023-07-18

**Authors:** Brianna J. McIntosh, Griffin G. Hartmann, Sean A. Yamada‐Hunter, Phillip Liu, Camille F. Williams, Julien Sage, Jennifer R. Cochran

**Affiliations:** ^1^ Cancer Biology Program Stanford University Stanford California USA; ^2^ Center for Cancer Cell Therapy, Stanford Cancer Institute Stanford University School of Medicine Stanford California USA; ^3^ Biophysics Program Stanford University Stanford California USA; ^4^ Department of Chemistry Stanford University Stanford California USA; ^5^ Department of Pediatrics Stanford University Stanford California USA; ^6^ Department of Genetics Stanford University Stanford California USA; ^7^ Stanford Cancer Institute Stanford University Stanford California USA; ^8^ Department of Bioengineering Stanford University Stanford California USA

**Keywords:** cancer progression, cytokines, IL‐11, ligand/receptor interaction, protein engineering

## Abstract

The cytokine interleukin (IL)‐11 has been shown to play a role in promoting fibrosis and cancer, including lung adenocarcinoma, garnering interest as an attractive target for therapeutic intervention. We used combinatorial methods to engineer an IL‐11 variant that binds with higher affinity to the IL‐11 receptor and stimulates enhanced receptor‐mediated cell signaling. Introduction of two additional point mutations ablates IL‐11 ligand/receptor association with the gp130 coreceptor signaling complex, resulting in a high‐affinity receptor antagonist. Unlike wild‐type IL‐11, this engineered variant potently blocks IL‐11‐mediated cell signaling and slows tumor growth in a mouse model of lung cancer. Our approach highlights a strategy where native ligands can be engineered and exploited to create potent receptor antagonists.

## 1 INTRODUCTION

Cytokines play an essential role in cell‐to‐cell signaling processes across nearly all systems in the body.^1^ The interleukin (IL)‐6 family of cytokines includes IL‐6, IL‐11, IL‐27, cardiotrophin‐like cytokine factor 1 (CLCF1), ciliary neurotrophic factor, leukemia inhibitory factor (LIF), and oncostatin M.^2^ These cytokines are comprised of four‐helical bundles and share the signaling coreceptor glycoprotein 130 (gp130) which is ubiquitously expressed throughout the body.^3^ Many of the IL‐6 family ligands and coreceptors have been targets of preclinical and clinical therapeutic campaigns for cancer and autoimmunity.^4^, ^5^, ^6^, ^7^, ^8^ Here, we focus on the pleiotropic cytokine, IL‐11, which has been implicated in a variety of diseases across tissue types.^9^

IL‐11 is a non‐glycosylated 178‐amino‐acid soluble cytokine with a molecular weight of ~19 kDa. It has been proposed to first bind to the IL‐11 receptor (IL‐11R), and along with gp130 forms a hexameric complex via two independent IL‐11‐gp130 binding sites.^10^ This complex promotes downstream Janus kinase (JAK) activation and signaling through signal transducer and activator of transcription 3 (STAT3), phosphoinositide 3‐kinase (PI3K), and extracellular signal‐regulated kinase (ERK).^11^, ^12^, ^13^ Increased IL‐11 signaling has been associated with cancer proliferation, metastasis, and chemoresistance,^14^, ^15^, ^16^, ^17^, ^18^ leading to the progression of gastric, colorectal, breast, and bone cancers among others.^19^, ^20^, ^21^, ^22^ IL‐11 has also been implicated in fibrotic remodeling that affects cancer‐associated fibroblasts as well as lung, liver, and heart fibrosis.^18^, ^23^, ^24^, ^25^, ^26^ As a result of these disease associations, the IL‐11 axis is currently being targeted by clinical‐stage antibodies for pulmonary fibrosis.^27^ Additionally, IL‐11 has been identified as a therapeutic target for lung adenocarcinoma.^28^, ^29^

Here, we used the IL‐11 ligand, which lacks binding to other IL‐6 family member receptors,^30^ as a starting point for developing a high‐affinity IL‐11R antagonist. Combinatorial methods enabled development of an engineered IL‐11 cytokine with ~70‐fold higher affinity to IL‐11R measured via yeast surface display as compared with wild‐type (WT) IL‐11. Introduction of two additional point mutations ablated binding to the gp130 coreceptor at two putative IL‐11 binding sites, resulting in an antagonist that disrupts the active signaling complex and decreases lung adenocarcinoma progression in a mouse tumor model.

## 2 RESULTS

### 2.1 Engineering agonist IL‐11 variants with increased affinity for IL‐11R

**FIGURE 1:**
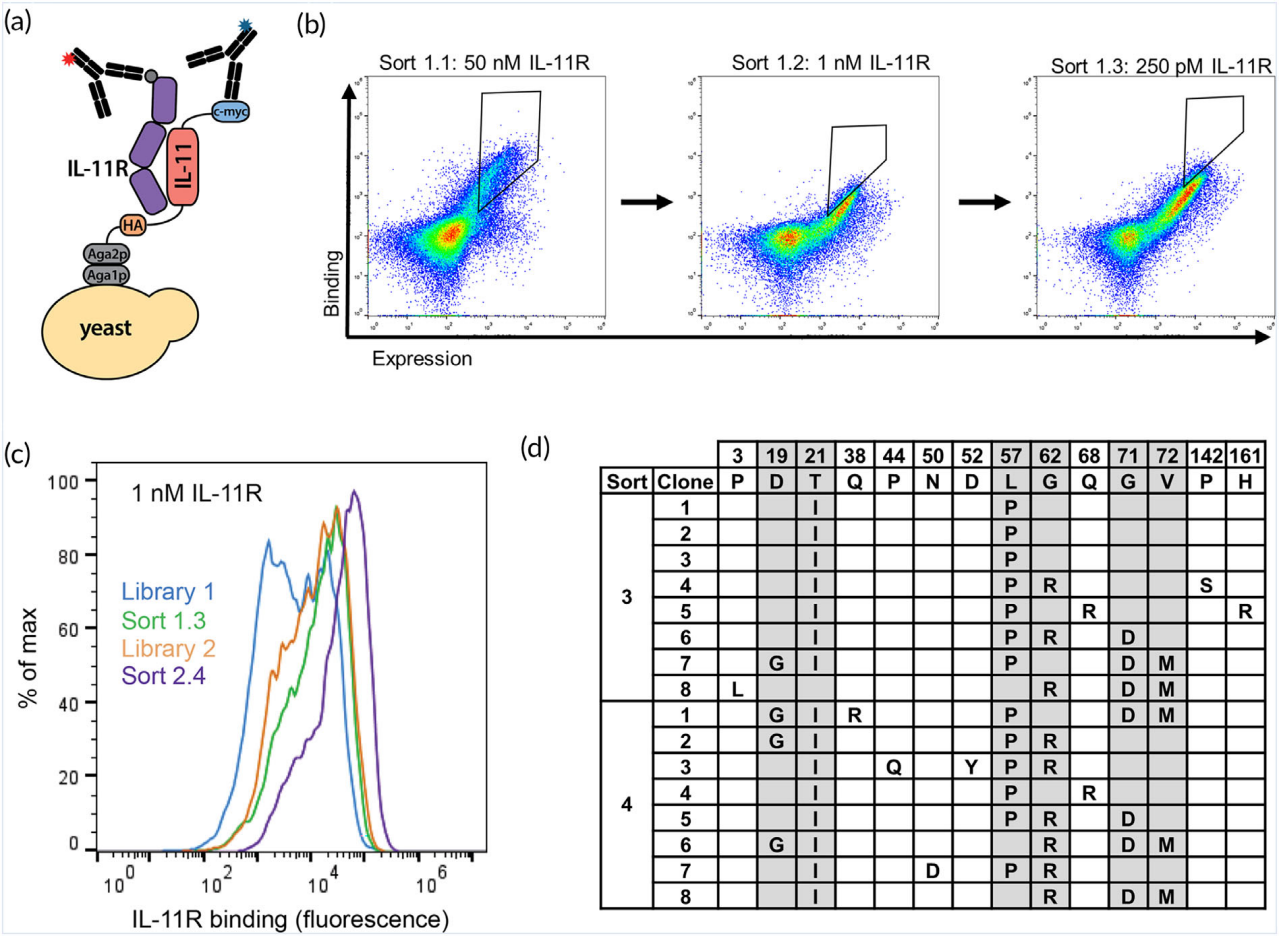
Screening yeast‐displayed IL‐11 libraries against human IL‐11 receptor. (a) Schematic of yeast surface‐displayed IL‐11 binding to soluble his‐tagged IL‐11R detected by fluorescent antibodies (blue: c‐myc tag for IL‐11 expression; red: IL‐11R binding detected by 6× histidine tag). (b) Three rounds of fluorescence activated cell sorting against indicated concentrations of IL‐11R. Polygons on cell dot plots indicate gates set to collect the yeast population that was used in the subsequent sort. (c) Improvement in binding of yeast from the final sort rounds to 1 nM IL‐11R compared with initial library 1 and 2. (d) Table of amino acid mutations from sequencing eight clones from post 2.3 and 2.4 sorts. Wild‐type IL‐11 amino acid residues are indicated along the top. Consensus mutations are highlighted in gray.

Combinatorial screening via yeast surface display was used to first identify human IL‐11 variants with improved affinity for the human IL‐11R extracellular domain. IL‐11 was displayed on the surface of yeast cells through genetic fusion to the agglutinin mating protein (Aga2p), which complexes with Aga1p contained in the yeast strain EBY100.^31^, ^32^ Yeast‐displayed IL‐11 expression was detected via flow cytometry using antibodies specific to a C‐terminal c‐myc tag, and IL‐11R binding was detected via antibodies against a 6× histidine tag (Figure 1a). A mutagenic library was created by error‐prone polymerase chain reaction (PCR) of the gene coding for human IL‐11,^33^ and the resulting DNA was electroporated into yeast strain EBY100 by homologous recombination to yield a library of ~5 × 10^7^ transformants.^34^ Three successive rounds of flow cytometric sorting were performed on yeast displaying individual IL‐11 variants, collecting clones with the strongest binding to soluble human IL‐11R during each sort (Figure 1b). Pools of isolated yeast clones were propagated in culture following each sort round, and IL‐11 genes contained in individual yeast from the final sort (post‐Sort 1.3) were sequenced. This analysis identified a consensus mutation (L57P) with an apparent binding affinity (*K*_d_) of 0.71 ± 0.17 nM, a ~10× improvement in human IL‐11R binding compared with wild‐type (WT) human IL‐11 (Figure S1a,b).

**FIGURE 2:**
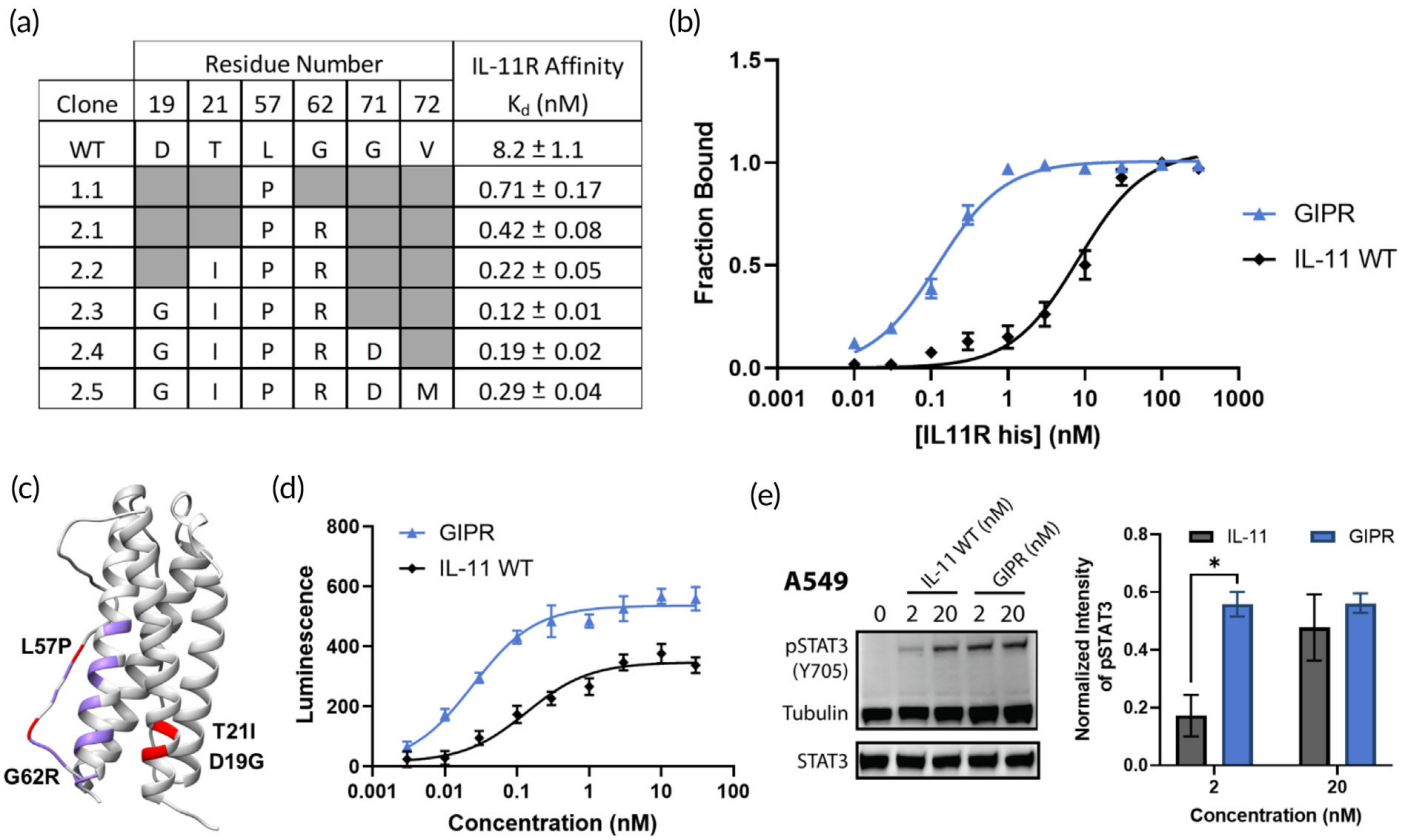
Characterizing IL‐11 variants with increased affinity for human IL‐11 receptor. (a) Table of individual variants along with their measured apparent affinities for IL‐11R compared with wild‐type (WT) IL‐11; variants are subsequently named after their mutations. (b) Dose‐dependent binding curve of human IL‐11R to yeast‐displayed IL‐11 GIPR and IL‐11 WT measured by flow cytometry. Points represent the fraction bound to IL‐11R of the expressing population ±SD, *n* = 3, curve fit by nonlinear regression. (c) IL‐11 crystal structure (PDB: 6O4O) with proposed IL‐11R binding residues (Site I) highlighted in purple and GIPR mutations highlighted in red. (d) Phospho‐STAT3 signaling measured by luminescence in HeLa reporter cells transduced with IL‐11R. IL‐11 GIPR EC_50_ = 24 ±4 pM; IL‐11 WT EC_50_ = 140 ± 30 pM. Points represent mean ± SD, *n* = 3, curve fit by nonlinear regression. (e) Western blot of A549 lysates for phospho‐STAT3 (Y705) with indicated concentration of IL‐11 WT or IL‐11 GIPR. Tubulin and STAT3 as loading controls. Right panel: Quantified pSTAT3/Tubulin levels, mean ± SD, *n* = 2. *p < 0.05 by unpaired *t*‐test. STAT3, signal transducer and activator of transcription 3.

A second‐generation IL‐11 library was created by DNA shuffling, based on the pool of variants isolated from the first library screen (post‐Sort 1.3), using staggered extension process (StEP) to recombine the mutations.^35^ In parallel, new mutations were introduced via error‐prone PCR. The resulting yeast‐displayed library of ~10^7^ yeast transformants was screened using four rounds of flow cytometric sorting of increasing stringency, again collecting the strongest human IL‐11R binding clones (Figure S2a). The final isolated library pool (post‐Sort 2.4) bound to IL‐11R more tightly than either of the initial libraries and the post‐Sort 1.3 pool (Figure 1c). DNA sequencing of post‐Sort 2.3 and 2.4 pools revealed six consensus mutations (D19G, T21I, L57P, G62R, G71D, and V72M) that appeared in multiple clones (Figure 1d). Yeast‐displayed IL‐11 mutants were individually tested for binding to soluble IL‐11R in an additive manner built upon L57P (‘P’) as ‘IP’, ‘IPR’, ‘GIPR’, ‘GIPRD,’ and ‘GIPRDM’ (Figures 2a and S2b). We found that the IL‐11 GIPR variant had the tightest apparent *K*_d_ of 0.12 ± 0.01 nM, a ~ 70x improvement over WT IL‐11 (Figure 2b). The L57P and G62R mutations fall within a region of IL‐11 (termed Site I) previously described to confer binding to IL‐11R (Figure 2c).^36^ To investigate the effect of IL‐11 affinity on downstream signaling, a HeLa reporter cell line containing the firefly luciferase gene under the control of a pSTAT3 inducible response element was transduced with full‐length IL‐11R. We show that the IL‐11 GIPR variant is an agonist cytokine that induces increased pSTAT3 signaling as compared with WT IL‐11 in the HeLa reporter cells and A549 human non‐small cell lung cancer cells (NSCLC; Figure 2d,e). We hypothesize that the increased maximal HeLa pSTAT3 signal induced by IL‐11 GIPR is due to increased receptor occupancy and residence time driven by the higher affinity compared with WT IL‐11.

### 2.2 Engineering high‐affinity IL‐11 variants with loss of binding to gp130 coreceptor

**FIGURE 3:**
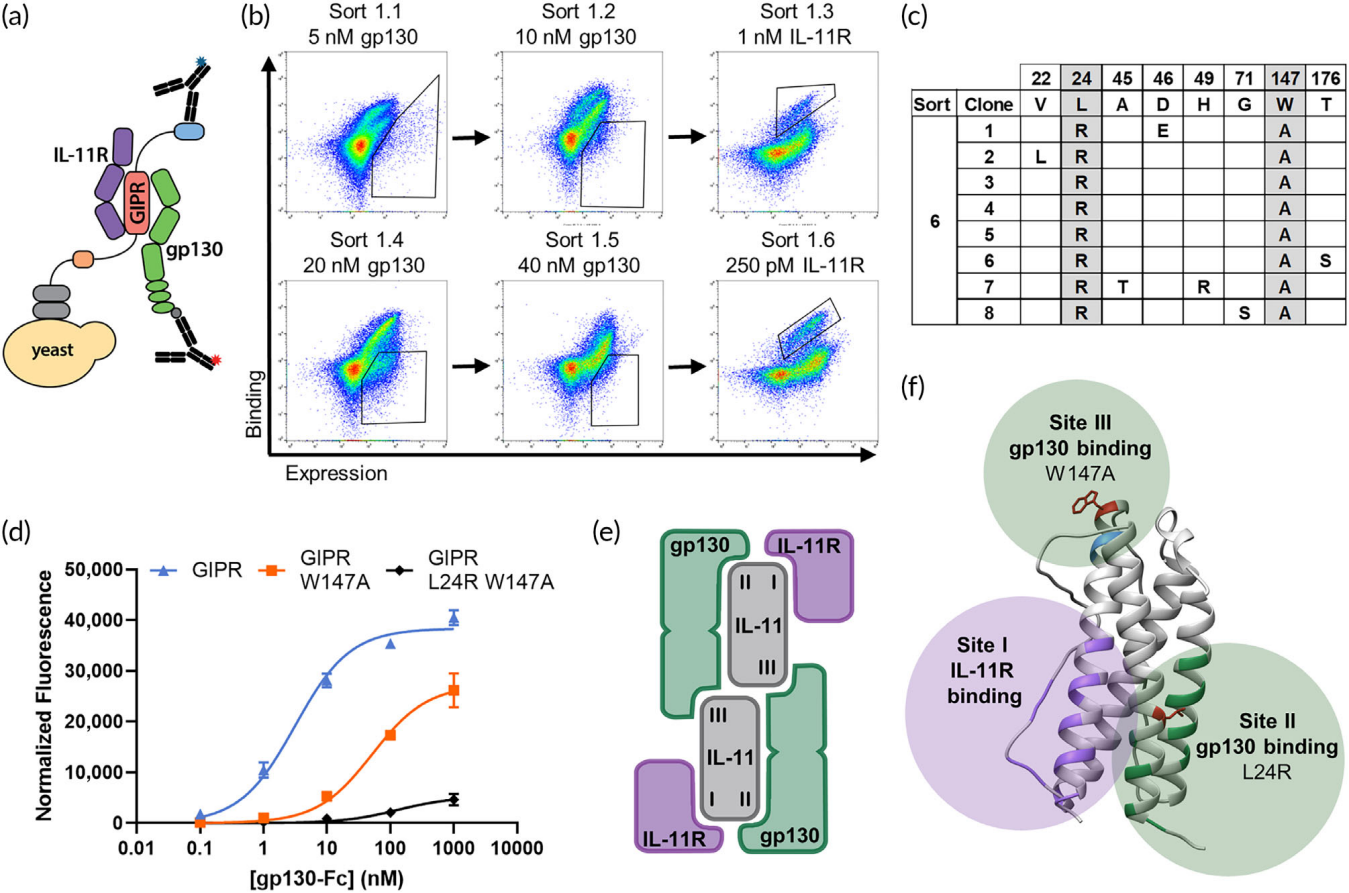
Engineering IL‐11 GIPR for loss of binding to gp130. (a) Schematic of trimeric complex of yeast surface‐displayed IL‐11 GIPR binding to soluble IL‐11R and gp130 detected by fluorescent antibodies (blue: c‐myc tag for IL‐11 GIPR expression; red: gp130 binding). (b) Yeast‐displayed IL‐11 GIPR library dot plots showing alternating two negative flow cytometry sorts against gp130 and one positive sort with IL‐11R at the indicated concentrations. Polygons indicate gates set to collect the yeast population that was used in the subsequent sort. (c) Table of amino acid mutations identified from sequencing eight clones after Sort 6. The wild‐type IL‐11 amino acid residue is indicated along the top. L24R and W147A are present in all eight clones. (d) Normalized binding of yeast‐displayed IL‐11 mutants to varying concentrations of gp130‐Fc with 100 nM IL‐11R added to all samples. Data points represent geometric mean of fluorescence of the expressing population ± SD, *n* = 3. (e) Schematic of IL‐11/IL‐11R/gp130 hexameric complex with IL‐11 Site I, III, and III labeled. (f) Crystal structure of IL‐11 (PDB: 6O4O) with purported Site I (IL‐11R binding residues) in purple and purported Site II and Site III (gp130 binding residues) in green and blue, respectively. L24R and W147A are shown in red.

Next, to develop an IL‐11R antagonist, we proceeded to create an IL‐11 cytokine that retains high‐affinity receptor binding but abrogates gp130 complex formation. IL‐11 is proposed to signal through a hexameric complex of IL‐11R and gp130. This complex forms through an initial binding event between IL‐11 and IL‐11R that predisposes the complex to bind with high affinity to gp130 and promote downstream signaling.^10^ Previously, a single point mutation, W147A, was reported to reduce binding of IL‐11 to gp130, converting IL‐11 from an agonist to an antagonist.^37^ Here, we found that IL‐11 GIPR W147A exhibited reduced, but still significant binding to gp130 when the variant was displayed on the surface of yeast and precomplexed with soluble IL‐11R (Figure 3a,d). Thus, we generated and screened an additional IL‐11 yeast library (~10^8^ transformants) to identify a more complete gp130 binding‐deficient mutant as an effective receptor antagonist. In this library, error‐prone PCR was used to introduce a low frequency of mutations to DNA templates encoding for IL‐11 GIPR and GIPR W147A in a 50/50 mixture. Two rounds of ‘negative’ screens were performed by flow cytometric sorting, where the yeast‐displayed IL‐11 library was precomplexed with a saturating concentration of IL‐11R before adding soluble gp130. The population of yeast clones that expressed IL‐11 but did not bind gp130 were collected in each of these sorts, followed by a ‘positive’ screen for IL‐11R binding‐competent clones to ensure that the overall IL‐11 structural integrity was not disrupted by mutations. This alternating ‘negative–positive’ screening strategy was repeated with increasing concentrations of gp130 and a reduced concentration of IL‐11R (Figure 3b). After six rounds of sorting, DNA sequencing of yeast clones identified two consensus mutations: L24R and the initial W147A mutation (Figure 3c). In further analysis, we showed that IL‐11 variants containing GIPR and GIPR W147A maintain significant gp130 binding whereas GIPR L24R W147A has minimal binding to gp130 (Figure 3d). Mapping the mutations onto the crystal structure of IL‐11 (PDB: 6O4O),^30^ we observed that L24R is located in a region known as Site II, one of two proposed gp130 binding sites on IL‐11, whereas W147A is located in the Site III region, the other proposed gp130 binding site (Figure 3e,f).^10^, ^38^ Thus, it is intriguing to postulate that the combination of the L24R and W147A mutations across two purported binding sites allows for more complete reduction of gp130 binding.

### 2.3 Engineering a human/mouse cross‐reactive IL‐11 variant

**FIGURE 4:**
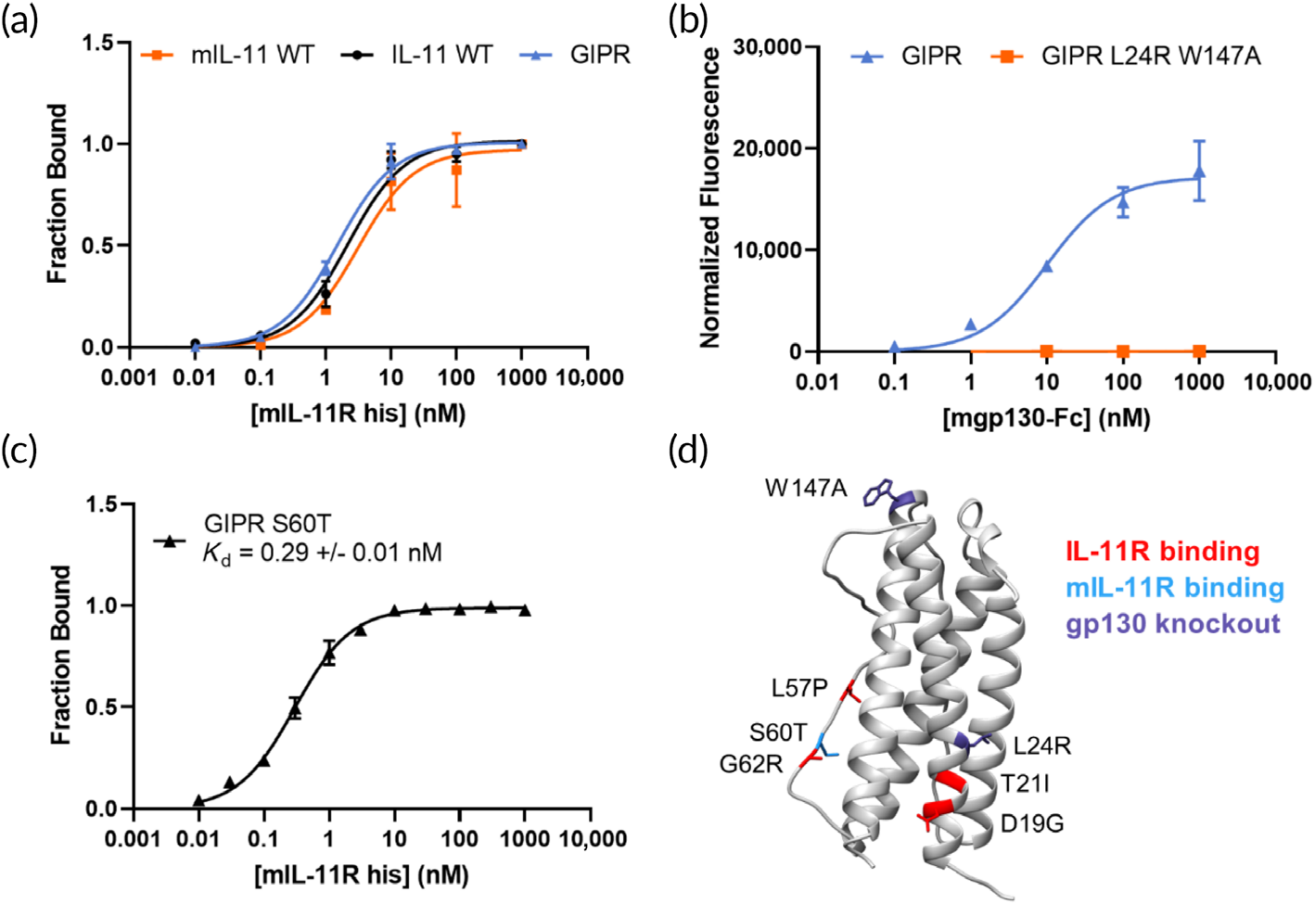
Affinity maturation of IL‐11 GIPR for mouse IL‐11R. (a) Binding of mIL‐11R to yeast‐displayed mIL‐11 wild‐type (WT; apparent *K*_d_ = 3.1 ± 0.8 nM); IL‐11 WT (apparent *K*_d_ = 2.2 ± 0.3 nM); or IL‐11 GIPR (apparent *K*_d_ = 1.5 ± 0.1 nM). Data points represent the fraction bound to mIL‐11R of the expressing population ± SD, *n* = 3, curve fit with non‐linear regression. (b) Binding of yeast‐displayed IL‐11 GIPR and GIPR L24R W147A pre‐complexed with 100 nM mIL‐11R to mouse gp130‐Fc. Data points are geometric mean of fluorescence of the expressing population ± SD, *n* = 3. (c) Binding curve of yeast‐displayed IL‐11 GIPR S60T to mIL‐11R by yeast surface display. Data points represent the fraction bound to mIL‐11R of the expressing population ± SD, *n* = 3, curve fit with non‐linear regression. (d) Crystal structure of IL‐11 (PDB: 6O4O) with the mutations that comprise enIL‐11 highlighted based on function in the indicated colors.

WT human IL‐11 and mouse IL‐11 (mIL‐11) bind to mouse IL‐11R (mIL‐11R) with similar low nanomolar affinity (Figure 4a). Our engineered IL‐11 GIPR variant did not show increased binding affinity to mIL‐11R (Figure 4a), indicating that affinity maturation against human IL‐11R did not consequentially confer tighter binding to mIL‐11R. Importantly, however, the L24R and W147A mutations also ablated mouse gp130 binding (Figure 4b), demonstrating that IL‐11 GIPR L24R W147A is effective in preventing formation of the mouse IL‐11/IL‐11R/gp130 hexameric complex.

To evaluate therapeutic efficacy more effectively in mouse lung cancer models, we sought to engineer an IL‐11 antagonist that has higher affinity for hIL‐11R and mIL‐11R. Using the yeast‐displayed GIPR library described above, we performed five rounds of flow cytometric sorting against decreasing concentrations of mIL‐11R (Figure S3a). Five consensus mutations were identified based on DNA sequencing of the post‐Sort 4 and 5 yeast pools (D41E, S60T, D79Y, H86Y, and G158E; Figure S3b). Desiring introduction of a minimally perturbing mutation, we selected to advance S60T, which like the L57P and G62R mutations lies in proximity to the proposed Site I IL‐11R binding region. When added to IL‐11 GIPR, S60T was shown to bind to mIL‐11R with an apparent *K*_d_ of 0.29 ± 0.01 nM, an ~10× improvement over WT mIL‐11 binding (Figure 4c).

Collectively, our final engineered variant of human IL‐11 (termed enIL‐11) has four mutations to improve the affinity to hIL‐11R (D19G, T21I, L57P, and G62R); one mutation to improve the affinity to mIL‐11R (S60T); and two mutations to ablate gp130 binding (L24R and W147A; Figure 4d). The enIL‐11 and WT IL‐11 proteins were recombinantly expressed for further characterization. Binding of soluble enIL‐11 to IL‐11R and mIL‐11R was measured via biolayer interferometry (BLI) with apparent *K*_d_ values of 0.41 ± 0.01 and 0.16 ± 0.01 nM, respectively, compared with 9.5 ± 0.3 and 11 ± 0.3 nM for WT IL‐11 (Figure S4a,b).

### 2.4 enIL‐11 inhibits cell signaling and slows tumor growth

**FIGURE 5:**
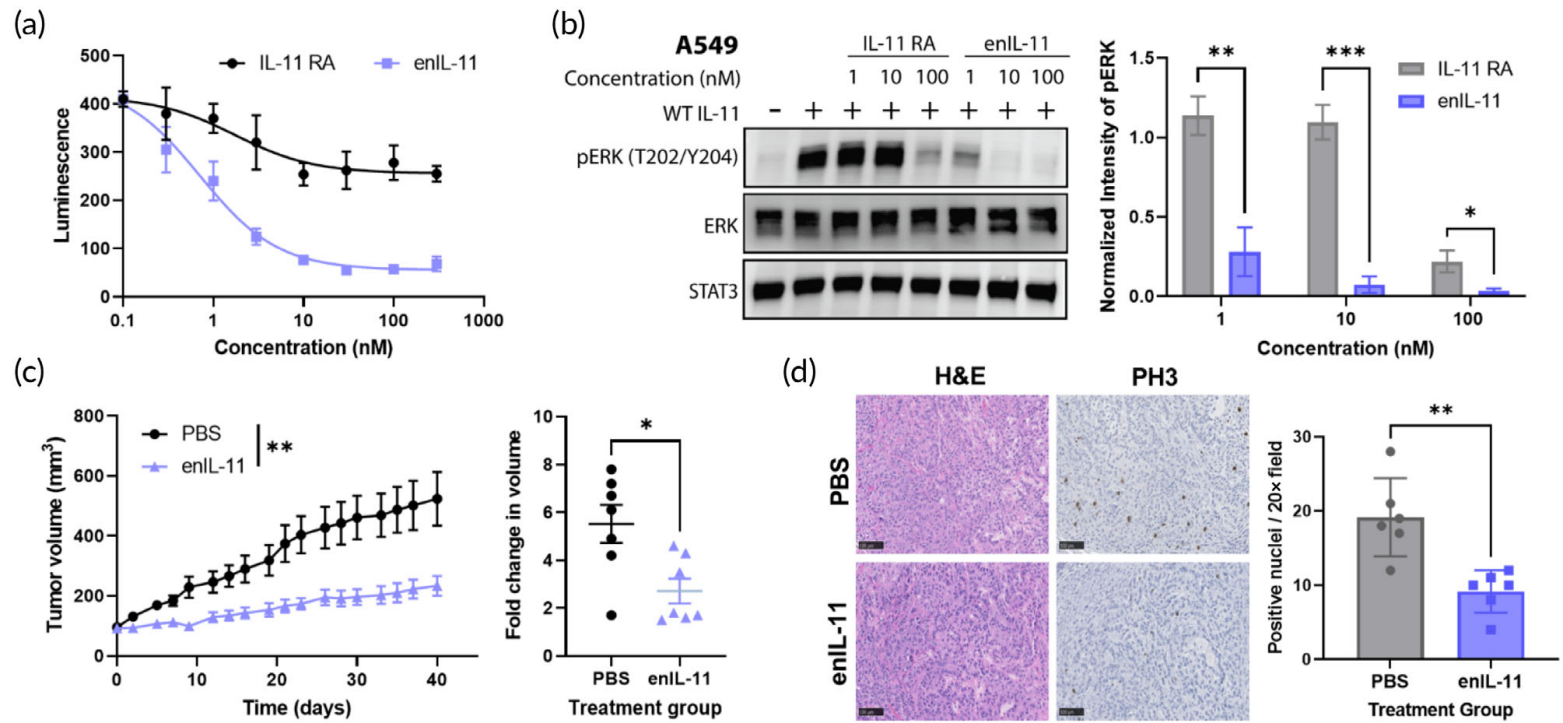
enIL‐11 inhibits IL‐11‐mediated cell signaling and slows tumor growth in vivo. (a) Phospho‐STAT3 signaling measured by luminescence in HeLa reporter cells transduced with IL‐11R and luciferase under control of a STAT3 inducible response element. 0.5 nM of wild‐type (WT) IL‐11 was added to all samples along with varying concentrations of WT IL‐11 L24R W147A mutations (IL‐11 RA) or enIL‐11. Data points represent mean ± SD, *n* = 3. (b) Western blot of A549 lysates probed for phospho‐ERK (T202/Y204) in response to 0 or 10 nM IL‐11 WT and the indicated concentrations of IL‐11 RA or enIL‐11. STAT3 and ERK as loading controls. Right panel: Quantified pERK/STAT3 levels normalized to the positive control, mean ± SD, *n* = 3. *p < 0.05, **p < 0.01, ***p < 0.001 by unpaired *t*‐test. (c) Nude mice bearing A549 flank tumors treated with 5 mg/kg of enIL‐11 (*n* = 7) or saline (*n* = 7) three times a week for 4 weeks. Points are mean ± *SE*. **p < 0.01 by two‐way analysis of variance (ANOVA). Final fold change in volume for each tumor plotted on the right. *p < 0.05 by unpaired *t*‐test. (d) Representative hematoxylin and eosin (H&E) staining and phospho‐histone H3 (PH3) immunohistochemistry for saline and enIL‐11‐treated tumors. Scale bars, 100 μm. Quantified positive nuclei were compared using unpaired *t*‐test (*n* = 6, **p < 0.01). Error bars represent ± SD. ERK, extracellular signal‐regulated kinase; PBS, phosphate‐buffered saline; STAT3, signal transducer and activator of transcription 3.

To directly parse the effects of improved receptor binding affinity, enIL‐11 was compared with a variant of WT human IL‐11 containing gp130‐disrupting mutations L24R and W147A (termed IL‐11 RA). The HeLa reporter cell line described above was used to interrogate the ability of the IL‐11 variants to compete with WT IL‐11 to block downstream pSTAT3 signaling. enIL‐11 and IL‐11 RA were added to the reporter cells at varying concentrations simultaneously with 0.5 nM of WT IL‐11. enIL‐11 more completely ablated pSTAT3 signaling with an IC_50_ of 0.7 ± 0.1 nM as compared with IL‐11 RA, which showed minimal signal inhibition (Figure 5a). Next, A549 cells were incubated with 20 nM of WT IL‐11 and increasing concentrations of IL‐11 RA and enIL‐11. Notably, enIL‐11 significantly decreased pSTAT3 levels at 1 and 10 nM of inhibitor compared with IL‐11 RA and was able to reduce pSTAT3 levels to baseline (Figure S5). This reduction in signaling was also seen for phospho‐ERK under the same conditions (Figure 5b).

The therapeutic efficacy of enIL‐11 was next tested in a mouse xenograft model of NSCLC using A549 cells engrafted into the flank of immunodeficient nude mice. Tumors were allowed to establish to an average volume of 100 mm^3^ before mice were binned into groups for treatment (Figure S6a). Mice were injected intraperitoneally with 1 or 5 mg/kg of enIL‐11 or a saline control three times a week for 4 weeks. No significant change in mouse body weight was observed between the treated and untreated groups (Figure S6b). Mice treated with 5 mg/kg saw significantly slower tumor growth compared with saline‐treated mice (Figure 5c). This reduction in tumor growth was observed in a dose‐dependent manner (Figure S6c,d). Tumors were excised and stained for the proliferation marker, phospho‐histone H3, and found to have a significant reduction in positive nuclei in the treated group (Figure 5d). Collectively, these results show that an engineered IL‐11 cytokine with mutations that increase IL‐11R binding affinity and ablate gp130 binding can function as a potent decoy ligand in cell culture and animal models.

## 3 DISCUSSION

IL‐11 has been shown to be upregulated by cancer cells and cancer‐associated fibroblasts in lung adenocarcinoma, leading to cancer progression.^18^, ^39^ In addition, increased IL‐11 mRNA expression in lung cancer patients is associated with worse overall survival.^40^ We used combinatorial protein engineering to identify a variant of IL‐11, containing four mutations (D19G, T21I, L57P, and G62R), that improved binding to IL‐11R. While two of these mutations are found outside of the previously defined Site I interface that binds to IL‐11R, it is possible the receptor binding site is broader than proposed or these residues could have effects on protein conformation via neighboring interactions. The other two consensus mutations G71D and V72M, while not found to be beneficial in the ‘GIPR’ context, could potentially have synergistic effects with different combinations of the four mutations and would be interesting to further explore in future studies. We similarly identified potential mutations to improve binding to mouse IL‐11R, and incorporated an additional putative Site I point mutation (S60T) to improve the apparent affinity of IL‐11 for mIL‐11R. Other mutations identified from our library screening efforts (D79Y and H86Y) might also further improve binding to mouse IL‐11R; however, these mutations significantly impacted recombinant protein expression and were not advanced. Thus, as the enIL‐11 cytokine binds to human IL‐11R with a tighter affinity as compared with mIL‐11R it is possible that its therapeutic efficacy in mouse tumor models will be underestimated due to less effective inhibition of endogenous mouse IL‐11 protein.

Conversion of the high‐affinity IL‐11 agonist cytokine into an antagonist was achieved by introducing two additional mutations, W147A and L24R, which significantly reduced binding to the gp130 coreceptor needed for a competent signaling complex. The W147A mutation was known from previous studies,^37^ while L24R was identified in our high‐throughput screen for variants that maintained binding to IL‐11R while reducing binding to gp130. Interestingly, these two mutations are positioned in disparate locations on IL‐11 (Figure 3f): W147A on Site III and L24R on Site II; regions which have been proposed to form binding sites for two distinct gp130 molecules.^36^ These two additional mutations along with the five mutations that improved affinity for mouse and human IL‐11R comprised our final enIL‐11 variant. A previous IL‐11 variant, termed IL‐11 mutein, was engineered using phage panning strategies (Figure S7a).^41^ We show that IL‐11 mutein binds to human IL‐11R with an apparent *K*_d_ of 1.6 ± 0.3 nM (~13× weaker than our GIPR variant), and also binds mouse IL‐11R at a similar affinity to WT IL‐11 (Figure S7b,c). IL‐11 mutein, which also contains the W147A mutation, has been shown to improve gastrointestinal cancer outcomes in mouse models.^20^, ^42^

Monoclonal antibodies are the most commonly used therapeutic modality to block receptor–ligand interactions.^43^ With regards to the IL‐11/IL‐11R signaling axis, an anti‐IL‐11R antibody is under evaluation in clinical trials as a treatment of pulmonary fibrosis. Additionally, an anti‐IL‐11 antibody has been evaluated in preclinical studies for liver and renal fibrosis among other fibrotic and inflammatory conditions.^25^, ^44^, ^45^ Antibodies benefit from avidity effects due to bivalency but can also induce receptor clustering and reduce diffusion due to their larger molecular weight. Engineered cytokines such as enIL‐11 seek to overcome the limitations of monovalency by taking advantage of natively high affinity and existing epitopes between ligand and receptor, presenting an opportunity to finely tune the cytokine effects, leveraging pleiotropy and the ability to bias signaling through structural changes.^46^ This effort has been achieved most prominently with IL‐2‐based biased agonists that are co‐receptor specific, leading to more tailored outcomes in modulating the activity of immune cells.^47^, ^48^ There has been further work building upon this concept to modulate other immune cytokines, such as interferon‐γ, to create agonists with biased signaling outcomes.^49^

Engineered cytokines with altered signaling capacities have also been explored with other members of the IL‐6 family, including IL‐6 variants with varying gp130 affinity and binding kinetics that affect downstream STAT signaling.^50^ Our lab has previously developed alternatives to antibodies in the form of ‘decoy’ ligands and receptors for various cancer subtypes and multiple members of the IL‐6 family.^7^, ^8^, ^51^, ^52^ Interestingly, when comparing the gp130 disrupting mutations found here in IL‐11 with previously identified mutations that disrupt CLCF1 binding to its coreceptors gp130 and LIFR, there are some similarities.^8^, ^53^ Residues previously found to disrupt gp130 binding in CLCF1 include a Y22C mutation, which falls on the same helical face as the L24R mutation in IL‐11 (Figure S8). In contrast to IL‐11, CLCF1 does not bind to two molecules of gp130, and instead relies on binding to gp130 and LIFR to form an active signaling complex. Our previous study found an F151A mutation that disrupts LIFR binding to CLCF1 and is located similarly to the W147A mutation on IL‐11 that disrupts the Site III binding of gp130 (Figure S8). This indicates that while CLCF1 and IL‐11 have only 28% sequence homology and leverage different receptor binding partners, similarities can be drawn in terms of their key coreceptor binding regions. Recently, an chimeric cytokine was created by grafting the LIFR binding residues of LIF onto Site III of IL‐11 to create a molecule that binds and signals through the non‐natural interaction of IL‐11R/LIFR/gp130.^54^ This work further demonstrates the modular nature of the IL‐6 family binding epitopes and the overlaps that key residues have in creating the same interactions on different scaffolds.

Higher affinity binding to IL‐11R, along with gp130 engagement, resulted in a cytokine agonist that more effectively induces cell signaling as measured by increased phospho‐STAT3 signaling. WT IL‐11 was approved as a treatment for thrombocytopenia to increase platelet production in 1997,^55^ thus, ‘super‐agonist’ forms of IL‐11 have potential to be utilized as a more effective alternative that could be pursued. Additionally, in future studies, it would be interesting to explore the effect that enIL‐11 has in other cancer types and fibrotic diseases that have shown a dependence on IL‐11. Moreover, IL‐11 has been shown to be upregulated by cancer‐associated fibroblasts in lung adenocarcinoma after treatment with chemotherapy as a chemoresistance mechanism.^18^ Therefore, the combination of enIL‐11 with chemotherapy could be used to proactively prevent chemoresistance onset.

## 4 CONCLUSIONS

We show that blocking IL‐11 with an engineered high‐affinity decoy ligand, enIL‐11, inhibits cell signaling and proliferation, and delays tumor progression in a mouse model of lung adenocarcinoma. As the roles for IL‐11 and its receptors become increasingly clear in diseases such as fibrosis and cancer, methods of inhibiting this signaling axis are of great interest. Our work with enIL‐11 further highlights a strategy where native ligands can be exploited to create potent cytokine‐derived receptor antagonists.

## 5 MATERIALS AND METHODS

### 5.1 Preparation of IL‐11 libraries

DNA encoding IL‐11 (residues Pro22 to Leu199) was cloned into the yeast display vector pCTcon2 (41843, Addgene) using *NheI* and *BamHI* restriction sites. This was used as the template for error‐prone PCR using Taq polymerase (50‐811‐694, Fisher Scientific), 50 mM MgCl_2_, and varying concentrations (1.25, 2.5, 5, and 10 μM) of dNTP analogs 2′‐dPTP (N‐2037, TriLink Biotechnologies) and 8‐oxo‐2′‐dGTP (N‐2034, TriLink Biotechnologies). The PCR products were pooled, amplified using Phusion polymerase, and purified by gel electrophoresis. The cDNA and linearized plasmid were electroporated into EBY100 yeast where they were assembled via homologous recombination. Library size was estimated to be 5 × 10^7^ via dilution plating. The second generation library was generated using the post‐Sort 1.3 pool as the template for error‐prone PCR and DNA shuffling using StEP.^35^ The extension protocol was run for 80 cycles with 94°C for 30 s and 55°C for 5 s. The error‐prone and StEP products were purified by gel electrophoresis and electroporated with linearized plasmid into EBY100 yeast. Library size was estimated to be 10^7^ by dilution plating. The GIPR/GIPR W147A library was created using Mutazyme II DNA polymerase (200550, Agilent) with 1 μg of DNA as template. The library size was estimated to be 10^8^.

### 5.2 Library screening

#### 5.2.1 IL‐11R screening

Yeast displaying high‐affinity IL‐11 variants were isolated using FACS on a Sony SH800 Sorter. The seven total rounds of screening against IL‐11R (Sino Biological, 10252‐H08H) were performed using equilibrium binding sorts by incubating the yeast library at room temperature in phosphate‐buffered saline (PBS) with 1 mg/mL bovine serum albumin (BSA) (BPBS) with the following concentrations of IL‐11R: 50 nM for Sort 1.1; 1 nM for Sort 1.2; 250 pM for Sort 1.3; 5 nM for Sort 2.1; 500 pM for Sort 2.2; 100 pM for Sort 2.3; and 50 pM for Sort 2.4. After the incubation with IL‐11R, yeast were washed with BPBS, pelleted, and resuspended in BPBS with a 1:5000 dilution of chicken anti‐c‐myc (A21281, Invitrogen) for 30 min at 4°C. Yeast were washed, pelleted, and resuspended with 1:500 dilutions of goat anti‐chicken AF488 (A11039, Fisher Scientific) and mouse anti‐his AF647 (MA1135A647, Thermo Scientific) for 15 min at 4°C. The labeled yeast were sorted by FACS with a minimum of 10× diversity coverage and the strongest binding clones based on the ratio of IL‐11R binding to c‐myc expression were collected. The sorted clones were propagated, induced, and used for the next round of screening. Plasmid DNA was recovered using a Zymoprep kit (D2001, Zymo Research Corp), transformed into DH10B electrocompetent cells, and isolated using a plasmid miniprep kit (K0503, Thermo Fisher). Sequencing was performed by MCLAB.

#### 5.2.2 gp130/IL‐11R screening

Yeast displaying the GIPR/GIPR W147A library were treated as described in the previous section with the following conditions: 100 nM IL‐11R and 5 nM gp130‐Fc (Sino Biological, 10974‐H03H‐20) for Sort 3.1; 100 nM IL‐11R and 10 nM gp130‐Fc for Sort 3.2; 1 nM IL‐11R for Sort 3.3; 100 nM IL‐11R and 20 nM gp130‐Fc for Sort 3.4; 100 nM IL‐11R and 40 nM gp130‐Fc for Sort 3.5; 250 pM IL‐11R for Sort 3.6. Incubations with IL‐11R were performed overnight, washed, and then gp130‐Fc was added for 3 h before staining as described above with goat anti‐human AF647 (SouthernBiotech, 2040‐31).

#### 5.2.3 mIL‐11R screening

Yeast displaying the GIPR/GIPR W147A library were treated as described in the previous section with the following conditions: 5 nM mIL‐11R (Sino Biological, 50,075‐M08H‐100) for Sort 4.1; 1 nM mIL‐11R for Sort 4.2; 250 pM mIL‐11R for Sort 4.3; 200 pM mIL‐11R for Sort 4.4; 150 pM mIL‐11R for Sort 4.5. Incubations with mIL‐11R were performed overnight, washed, and then stained as described above.

### 5.3 Yeast‐surface display binding assays

Yeast‐displayed IL‐11 variants were incubated with varying concentrations of IL‐11R or mIL‐11R overnight at room temperature to reach equilibrium, washed with BPBS, pelleted, and stained with a 1:5000 dilution of chicken anti‐c‐myc for 30 min at 4°C. The yeast were washed with BPBS, pelleted, and stained with a 1:500 dilution of goat anti‐chicken AF488 and mouse anti‐his AF647 for 15 min at 4°C. The samples were washed and analyzed by flow on a Accuri C6 cytometer (BD Biosciences) and data was analyzed using FlowJo. To characterize binding to gp130, yeast‐displayed IL‐11 variants were incubated with 100 nM of IL‐11R or mIL‐11R for a minimum of 3 h, washed with BPBS, pelleted, and resuspended with varying concentrations of gp130‐Fc. The gp130‐Fc was detected with a 1:500 dilution of goat anti‐human AF647.

### 5.4 Protein expression

cDNA of IL‐11 with an N‐terminal fusion of 6× histidine tag, thioredoxin, and tobacco etch virus (TEV) protease cleavage site was cloned into the pET28 plasmid with an inducible lac promoter and amplified in DH10B cells. Purified plasmid was transformed into BL21 cells. Cells at an optical density (OD_600_) between 0.5–0.8 were induced with 0.1 mM isopropyl‐B‐o‐thiogalactoside (Gold Biotechnology, I2481C25) in LB overnight at 30°C. Induced cells were pelleted and resuspended in a phosphate buffer with 10 mM imidazole (Sigma‐Aldrich, I2399‐500G). The cell resuspension was frozen at −80°C for 20 min and then allowed to thaw at room temperature. The resuspension was sonicated for 10 min and then centrifuged for 20 min at 10,000 rpm. The supernatant was purified using cobalt beads (H‐310‐50, Gold Biotechnology). Protein was concentrated and buffer‐exchanged into PBS using Amicon Ultra‐15 centrifugal filter units (UFC901024, Millipore‐Sigma, 10 kDA cutoff). Protein was cleaved overnight at 4°C with TEV protease (P8112S, NEB) and purified with cobalt beads. The cleaved IL‐11 was collected in the flowthrough and buffer exchanged into PBS. Concentration was measured using a NanoDrop 2000 (Thermo Fisher).

### 5.5 Biolayer interferometry

Biotinylated IL‐11R‐Fc (ILR‐H82F5, Acro) or mIL‐11R‐mFc (7405‐MR, R&D Systems) was loaded onto streptavidin‐coated biosensors (19‐5019, ForteBio) or antimouse Fc biosensors (18‐5088, ForteBio) at 1.25 μg/mL in kinetics buffer (18‐1105, Sartorius). Three‐fold serial dilutions of WT IL‐11 or enIL‐11 were made in kinetics buffer. Baseline measurement was performed in kinetics buffer alone, then association in the WT IL‐11 or enIL‐11 conditions followed by dissociation in kinetics buffer alone. Data were collected with an Octet (ForteBio) and analyzed using the Octet Data Analysis software. Binding to enIL‐11 was performed by Antibody Solutions.

### 5.6 HeLa STAT3 luciferase reporter cell assays

A STAT3 luciferase reporter HeLa stable cell line (SL‐0003‐FP, Signosis) was transfected with full‐length IL‐11R and cultured in Dulbecco's Modified Eagle Medium (DMEM) +10% fetal bovine serum (FBS) +100 μg/mL of Hygromycin B (Fisher Scientific, 10‐687‐010). Cells were seeded 16 h before the assay into 96‐well plates at 1.5 × 10^4^ cells/well. The culture medium was switched to DMEM with 0.1% FBS for 2 h prior to adding the cytokines. Dilutions of the cytokines were made in DMEM with 0.1% FBS. For agonism studies, varying concentrations of IL‐11 or GIPR were added. For antagonism studies, 0.5 nM of IL‐11 was added to all conditions simultaneously with varying concentrations of inhibitor. Cytokines were incubated on the cells at 37°C for 4 h. To quantify luciferase activity, cells were lysed with 20 μL of Lysis Buffer (E297A, Promega) for 10 min and then transferred to a white bottom 96‐well plate. Luciferase assay substrate (E151A, Promega) was resuspended in luciferase assay buffer (E152A, Promega) and 100 μL was added to each well. Luminescence was measured on a BioTek Synergy H4 plate reader.

### 5.7 Phospho‐STAT3 and phospho‐ERK signaling assays

A549 cells were seeded the day before the assay in a 12‐well plate at 1.5 × 10^5^ cells per well. Cells were starved in serum‐free media for 2 h. For phospho‐ERK assays, 10 μM U0126 (9903S, Cell Signaling Technology) was added to reduce baseline signaling levels. For agonism assays, IL‐11 or GIPR was added at indicated concentrations. Cytokines were diluted in serum‐free media with 0.1% BSA. For antagonism assays, IL‐11 was added at 20 nM simultaneously with the indicated concentration of IL‐11 RA or enIL‐11. Cytokines were added to the serum‐starved cells for 20 min at 37°C. Cells were washed twice with cold PBS and then 100 μL of RIPA Lysis and Extraction buffer (89900, Fisher Scientific) with phosphatase inhibitor (P5726, Sigma‐Aldrich) and protease inhibitor (P8340, Sigma‐Aldrich) were added to each well for 10 min. Protein lysate concentrations were quantified using a bicinchoninic acid (BCA) assay (23225, Fisher Scientific) and equivalent amounts of protein (15–20 μg) were ran on a (polyrylamide gel electrophoresis) PAGE gel. Samples were transferred to a nitrocellulose membrane (IB301031, Thermo Fisher) with an iBlot dry blotting system. Blots were blocked overnight in 1x tris‐buffered saline + 0.1% Tween 20 (TBST) with 5% BSA. STAT3 signaling blots were stained with 1:4000 phospho‐stat3 (pY705) rabbit antibody (9145S, Fisher Scientific) and 1:5000 anti‐β‐tubulin mouse antibody (50‐103‐0148, Fisher Scientific), or 1:5000 Stat3 (D3Z2G) rabbit antibody (NC0969631, Fisher Scientific) in TBST +5% BSA for 2 h at room temperature. ERK signaling blots were stained with 1:5000 phospho‐p44/42 MAPK (Erk1/2) (Thr202/Tyr204; 9101, Cell Signaling Technology) and 1:5000 STAT3 antibody or 1:5000 p44/42 MAPK (Erk1/2; 9102, Cell Signaling Technology). Blots were washed three times for 5 min each with TBST. Secondary staining was done with 1:5000 HRP‐conjugated anti‐rabbit (711‐035‐152, Jackson ImmunoResearch) and anti‐mouse antibodies (715‐035‐150, Jackson ImmunoResearch) in TBST +5% BSA for 1 h at room temperature before being washed again. Blots were incubated with SuperSignal West Femto Maximum Sensitivity Substrate (34095, Fisher Scientific) and chemiluminescence was detected using a ChemiDoc XRS System (Bio‐Rad). Images were quantified using ImageJ.

### 5.8 In vivo tumor model

A549 cells (10^6^) mixed 1:1 with Matrigel for a total volume of 100 μL (CB‐40230, Fisher Scientific) were injected subcutaneously in the right flank of 8‐week‐old nude mice (NU/J 002919, The Jackson Laboratory). Tumors were allowed to grow to an average of 100 mm^3^ before being stratified into treatment groups. enIL‐11 (GIPR RA S60T) in saline was injected intraperitoneally three times a week at 1 and 5 mg/kg. Saline alone was used as the control. The tumor volume was measured by digital calipers three times per week and calculated using volume = (length) × (width) × 0.5 (width). Tumors were excised at the end of the study and fixed in 10% buffered formalin for 24 h and then stored in 70% ethanol before paraffin embedding. Immunohistochemistry staining was done by HistoTec Labs.

### 5.9 Ethics statement

Mice were maintained and animal experiments performed in accordance with policies approved by the Stanford University Administrative Panel on Laboratory Animal Care (protocol no. 33187).

## AUTHOR CONTRIBUTIONS

**Brianna J. McIntosh:** Conceptualization (equal); formal analysis (lead); investigation (lead); writing—original draft (equal). **Griffin G. Hartmann:** Investigation (supporting). **Sean A. Yamada‐Hunter:** Methodology (equal). **Phillip Liu:** Investigation (supporting). **Camille F. Williams:** Investigation (supporting). **Julien Sage:** Supervision (supporting); writing—review and editing (supporting). **Jennifer R. Cochran:** Conceptualization (equal); supervision (lead); writing—original draft (equal).

## CONFLICT OF INTEREST STATEMENT

Brianna J. McIntosh and Jennifer R. Cochran are inventors on a patent application related to this work that is owned by the Stanford University. Jennifer R. Cochran is a cofounder and equity holder of the Combangio, Inc. (now Kala Pharmaceuticals), xCella Biosciences (now OmniAb), and Red Tree Venture Capital; an equity holder of Aravive, Inc., and a member of the Board of Directors of OmniAb,  Revel Pharmaceuticals, Biograph 55, and Excellergy Therapeutics; and is a Board Observer at Tachyon Therapeutics and Acrigen Biosciences. All other authors declare that they have no competing interests.

## Supporting information


**Data S1:** Supporting information.

## Data Availability

The data that support the findings of this study are available from the corresponding author upon reasonable request.
