# Supplementary material for: An engineered interleukin‐11 decoy cytokine inhibits receptor signaling and proliferation in lung adenocarcinoma
Source: Bioeng Transl Med. 2023 Jul 18;8(6):e10573. doi: 10.1002/btm2.10573 (PMC10658506; doi:10.1002/btm2.10573)
Supplement: Supplementary file 1 — Data S1: Supporting information. [file BTM2-8-e10573-s001.docx]

Supporting Information

**An engineered interleukin-11 decoy cytokine inhibits receptor signaling and proliferation in lung adenocarcinoma**

Brianna J. McIntosh^1^, Griffin G. Hartmann^1^, Sean A. Yamada-Hunter^2^, Phillip Liu^3^, Camille F. Williams^4^, Julien Sage^5,6,7^, Jennifer R. Cochran^1,7,8^

1 Cancer Biology Program, Stanford University, Stanford, California 94305, USA

2 Center for Cancer Cell Therapy, Stanford Cancer Institute, Stanford University School of Medicine, Stanford, CA 94305, USA.

3 Biophysics Program, Stanford University, Stanford, California 94305, USA

4 Department of Chemistry, Stanford University, Stanford, California 94305, USA

5 Department of Pediatrics, Stanford University, Stanford, CA 94305, USA

6 Department of Genetics, Stanford University, Stanford, CA 94305, USA

7 Stanford Cancer Institute, Stanford University, Stanford, CA 94305, USA

8 Department of Bioengineering, Stanford University, Stanford, California 94305, USA

Contact information: [jennifer.cochran@stanford.edu](mailto:jennifer.cochran@stanford.edu); 650-724-7808

443 Via Ortega, Stanford, CA 94305


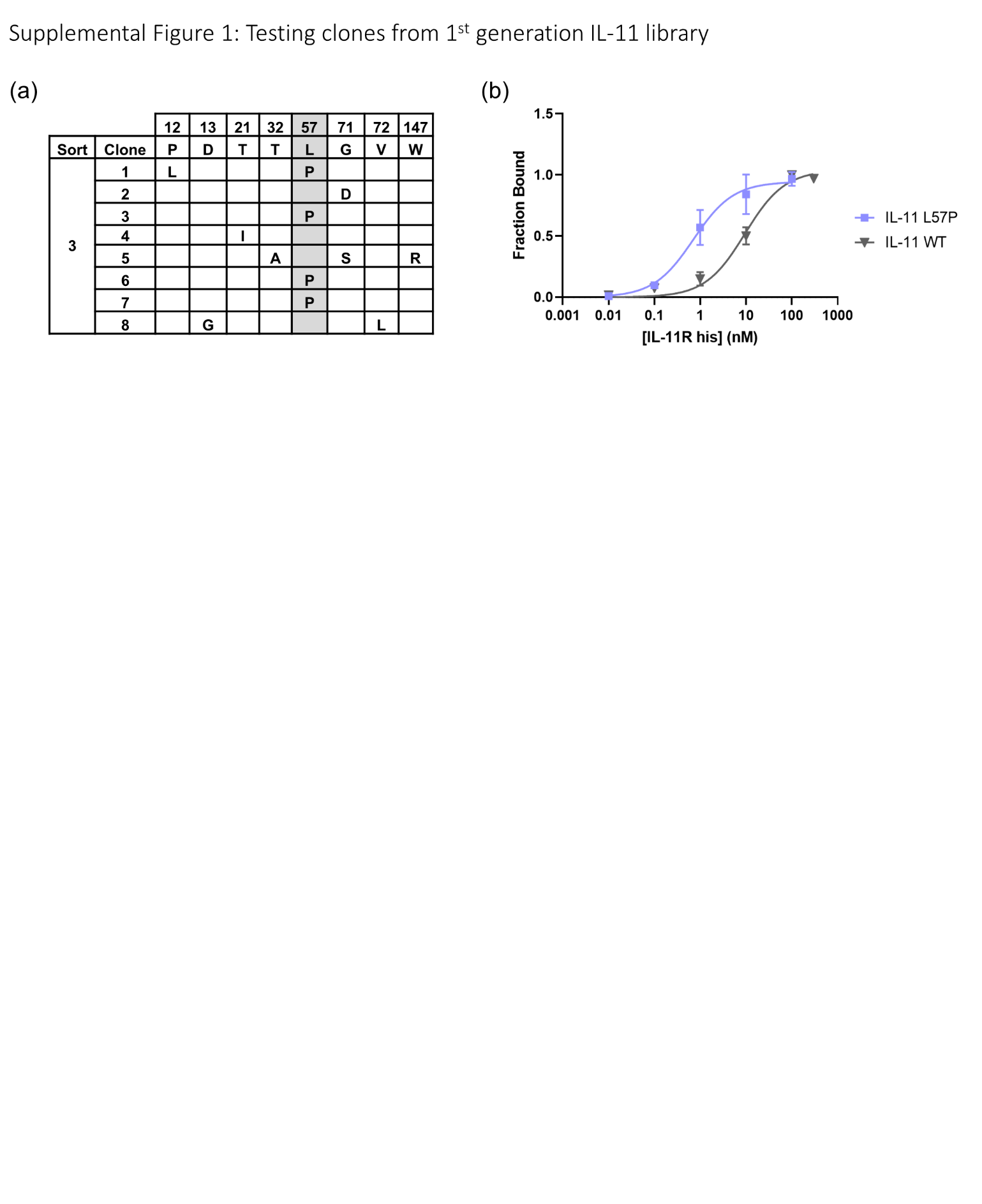


**Figure S1: Characterization of first generation IL-11 library clones**. (a) Table of amino acid mutations identified from sequencing eight clones after sort 1.3 of yeast-displayed Library 1, generated by error-prone PCR. The wild-type (WT) IL-11 amino acid residue is indicated along the top. L57P, highlighted in gray, is seen in 4 out of 8 clones. (b) Binding curve of yeast-displayed IL-11 L57P compared to WT IL-11 as measured by flow cytometry. Data points represent fraction bound to IL-11R of the expressing population +/- SD, n = 3, curve fit by non-linear regression.


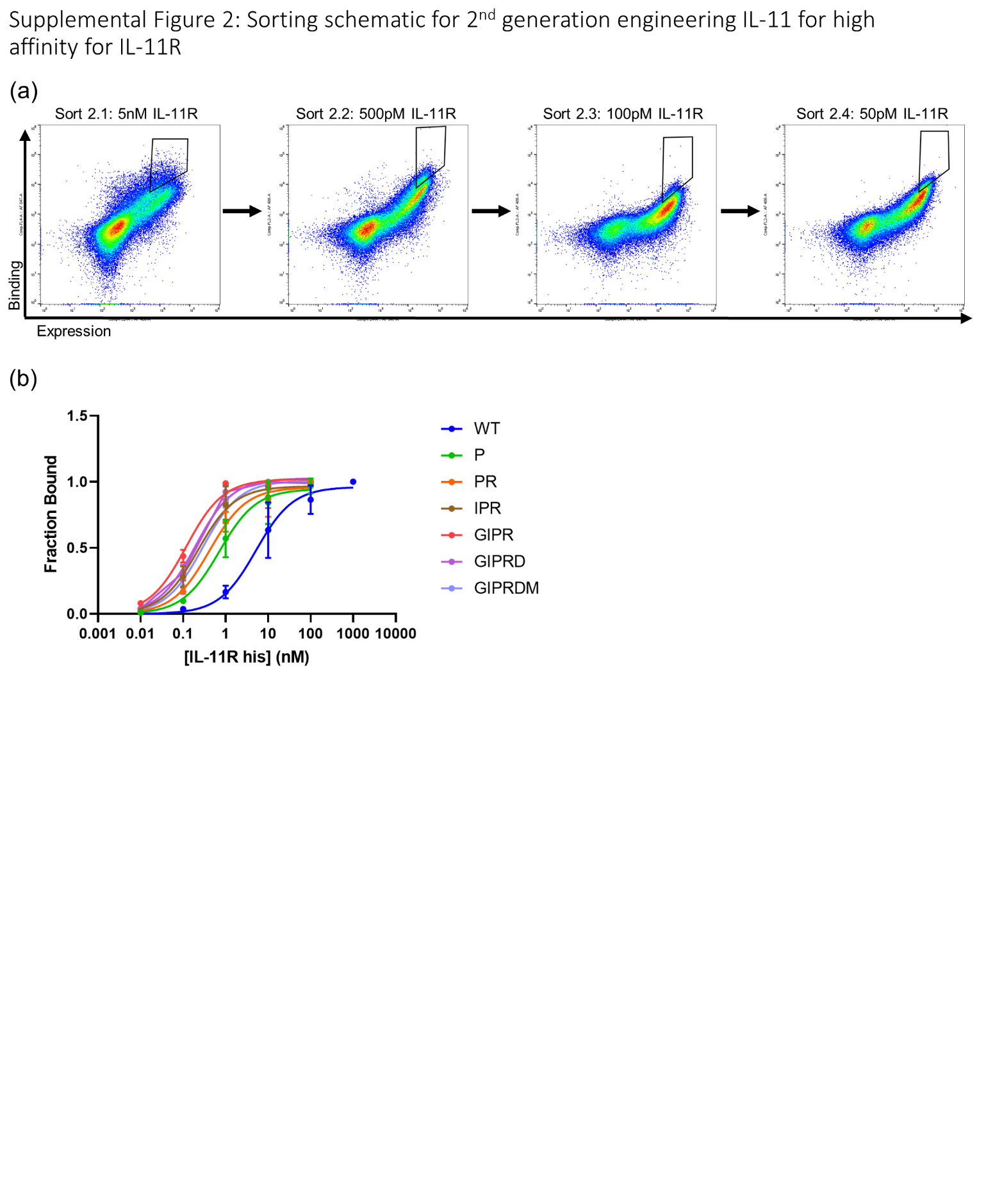


**Figure S2:** **Second generation IL-11 library sorting and characterization.** (a) Dot plots representing four rounds of fluorescence activated cell sorting of yeast-displayed IL-11 library 2 (generated by DNA shuffling and error-prone PCR) against indicated concentrations of IL-11R. Polygons indicate gates set to collect the yeast population that was used in the subsequent sort. (b) Dose dependent IL-11R binding curves of yeast-displayed variants and WT IL-11 measured by flow cytometry. Data points represent fraction bound to IL-11R of the expressing population +/- SD, n = 3, curve fit by non-linear regression.


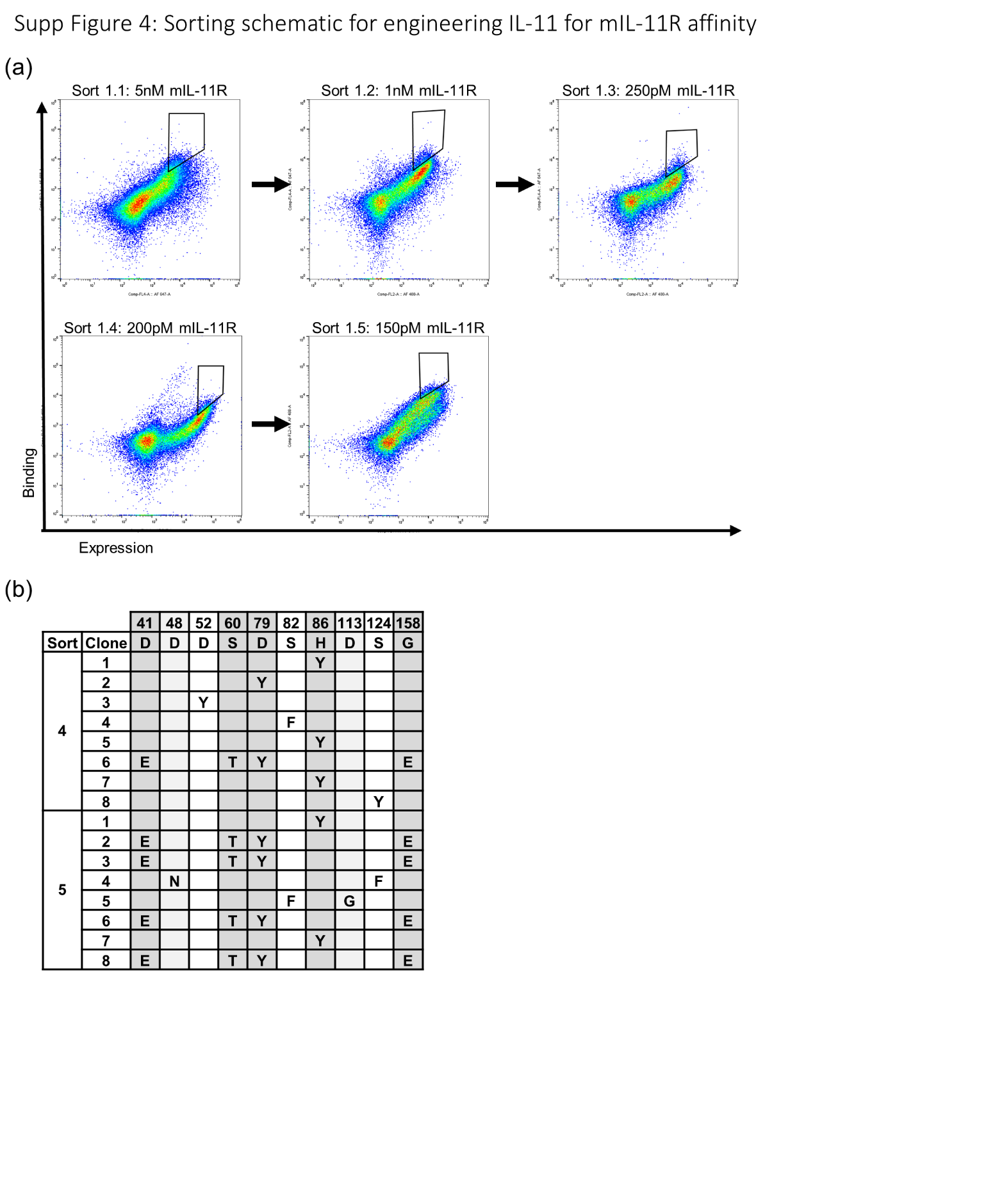


**Figure S3: Affinity maturation of IL-11 GIPR for mouse IL-11R.** (a) Dot plots representing five rounds of fluorescence activated cell sorting of yeast-displayed IL-11 library against indicated concentrations of mIL-11R. Polygons indicate gates set to collect the yeast population that was used in the subsequent sort. (b) Table of amino acid mutations identified from sequencing eight clones from post fourth and fifth round sorts. The wild-type IL-11 amino acid residue is indicated along the top. Consensus mutations are highlighted in gray.

**
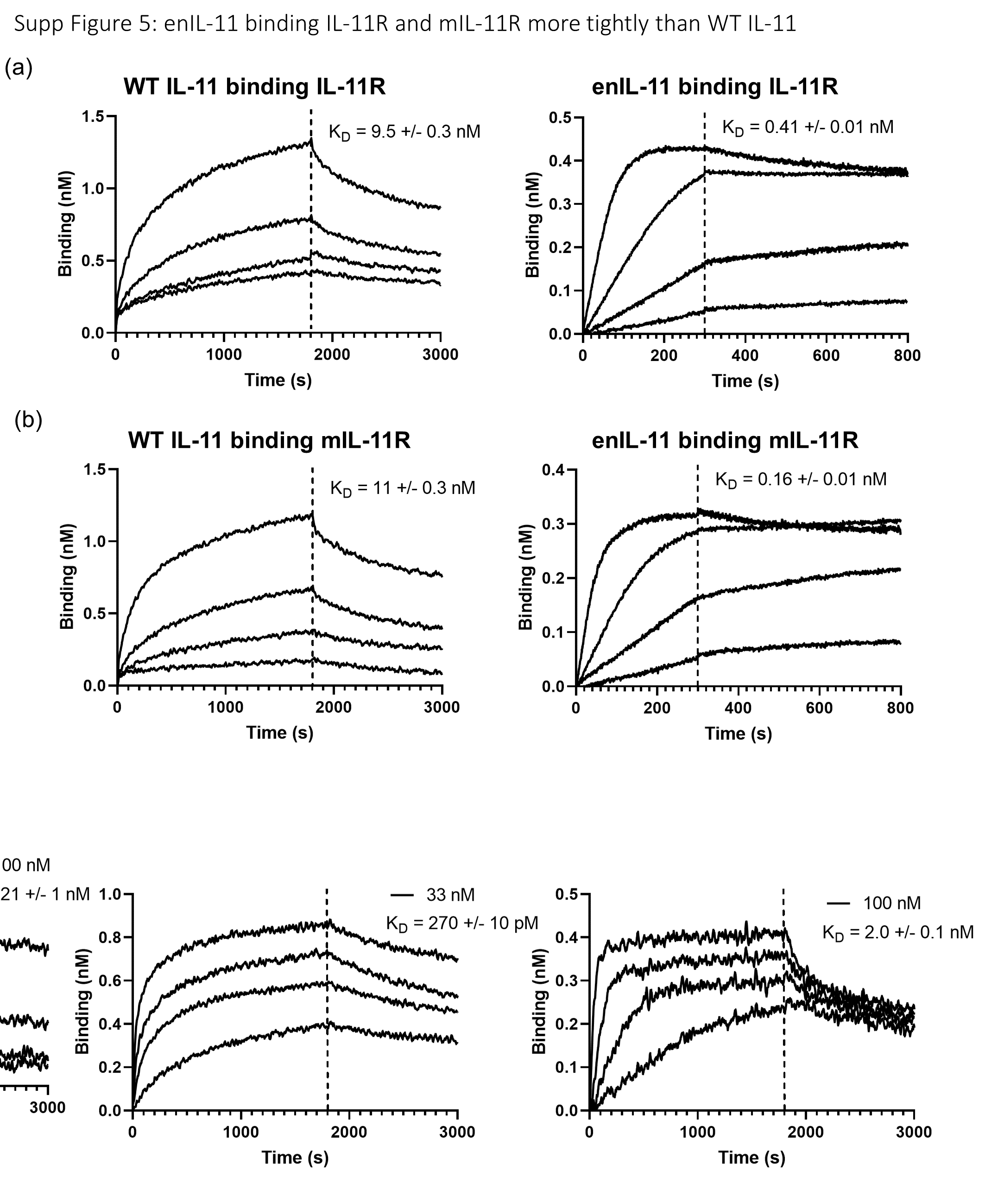
Figure S4: enIL-11 binds IL-11R and mouse IL-11R more tightly than WT IL-11 via biolayer interferometry (BLI).** (a) Three-fold serial dilutions from 100 nM for WT IL-11 and 33 nM for enIL-11 binding to IL-11R. (b) Three-fold serial dilutions from 100 nM for WT IL-11 and 33 nM for enIL-11 binding to mIL-11R.


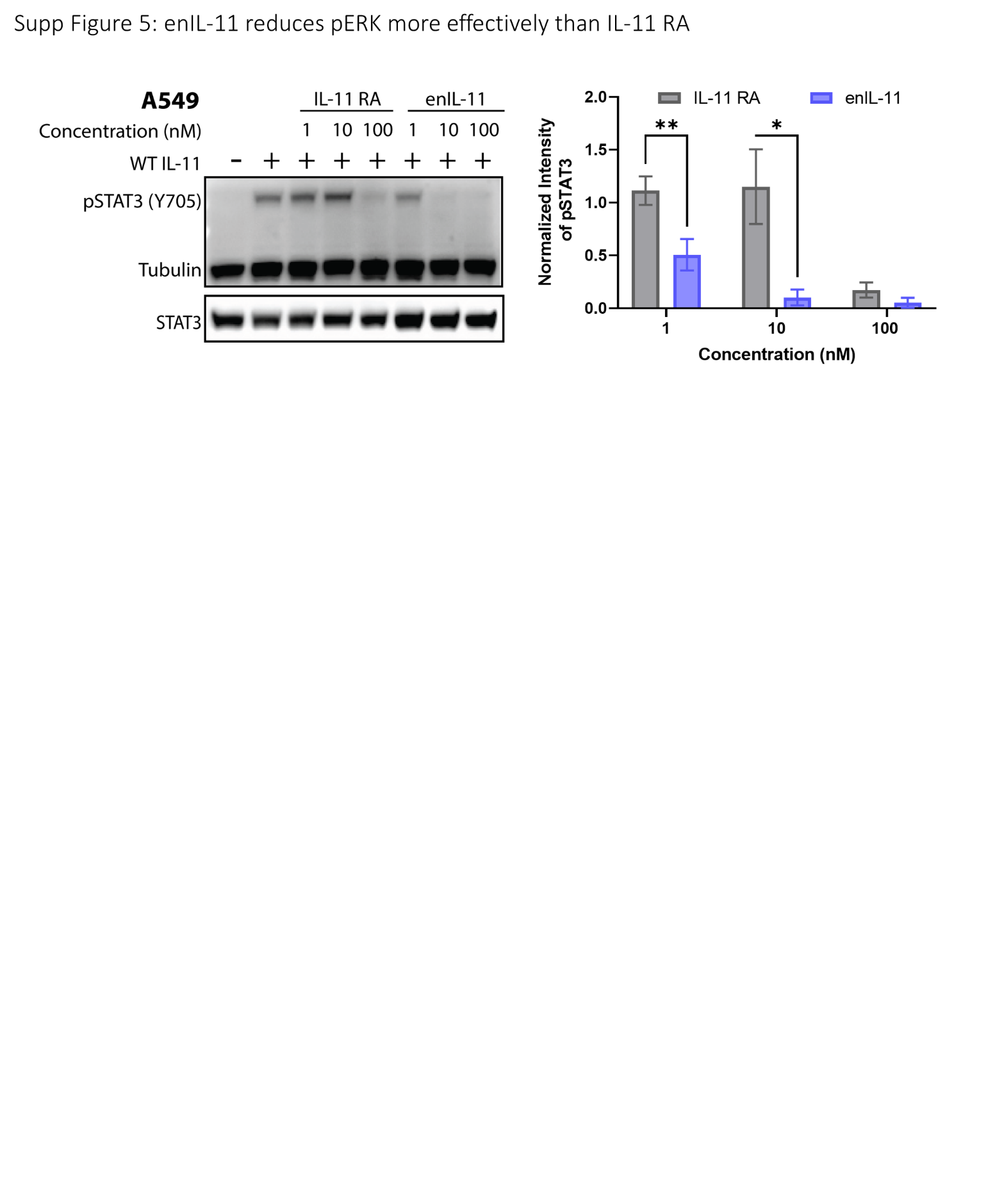


**Figure S5: enIL-11 reduces phospho-STAT3 signaling on A549 cells.** Western blot of A549 lysates probed for phospho-STAT3 (Y705) in response to 0 or 20 nM WT IL-11 and the indicated concentrations of IL-11 RA or enIL-11. Tubulin and STAT3 as loading controls. Right panel: Quantified pSTAT3/Tubulin levels normalized to the positive control, mean +/- SD, n = 3. *P < 0.05, **P < 0.01 by unpaired t-test.


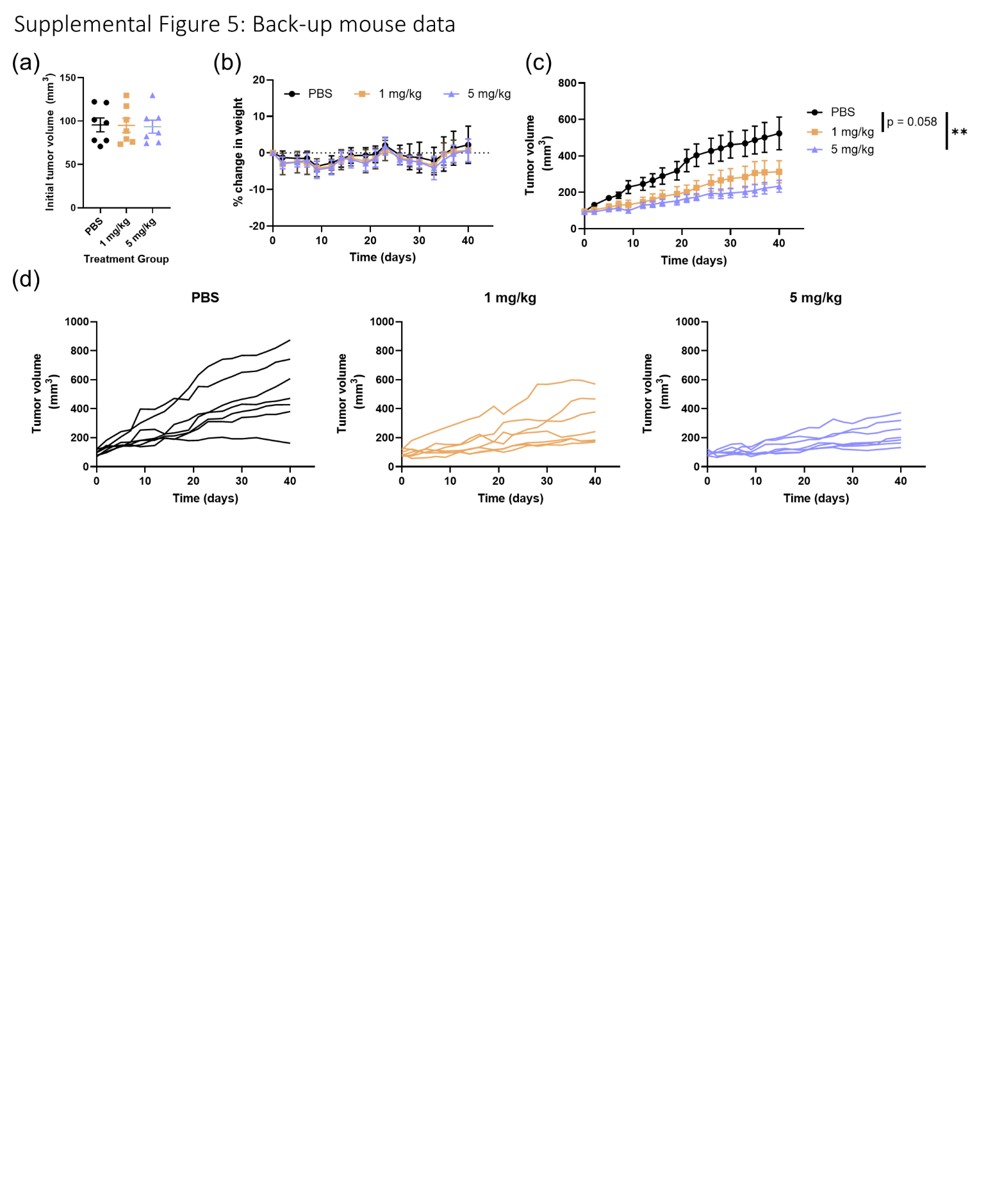


**Figure S6: enIL-11 reduces A549 tumor growth in dose dependent manner**. (a) Initial tumor volumes of the three treatment groups. (b) Change in weights shown as a percentage of starting weight for the three treatment groups. Data points represent mean +/- SD. (c) Nude mice with A549 flank tumors treated with 5 mg/kg of enIL-11 (n = 7), 1 mg/kg of enIL-11 (n = 7), or saline (n = 7) three times a week for four weeks. Data points represent mean +/- SE. **P < 0.01 by two-way ANOVA. (d) Individual volume traces for the three treatment groups over the course of the study.


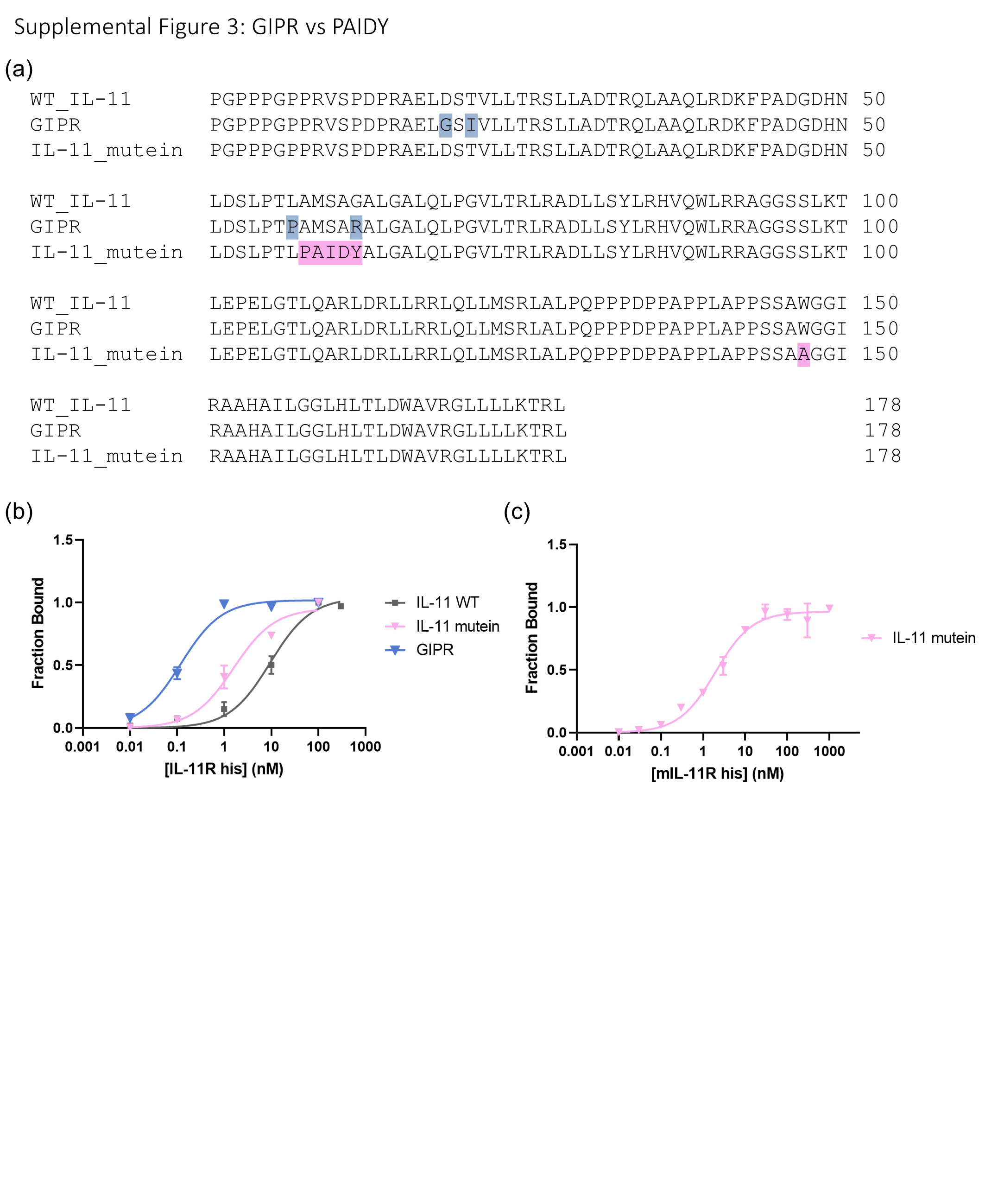


**Figure S7: Comparison of IL-11 GIPR and previously engineered IL-11 mutein**. (a) Sequence alignment of wild-type (WT) IL-11, IL-11 GIPR, and IL-11 mutein, with differences highlighted. (b) Dose dependent binding curve of human IL-11R to yeast-displayed IL-11 mutein, WT IL-11, and IL-11 GIPR as measured by flow cytometry. IL-11 mutein apparent K_d_ = 1.6 +/- 0.3 nM. Data points represent fraction bound to IL-11R of the expressing population +/- SD, n = 3, curve fit by non-linear regression. (c) Dose dependent binding curve of mouse IL-11R to yeast-displayed IL-11 mutein as measured by flow cytometry. IL-11 mutein apparent K_d_ = 2.0 +/- 0.2 nM. Data points represent fraction bound to mouse IL-11R of the expressing population +/- SD, n = 3, curve fit by non-linear regression.


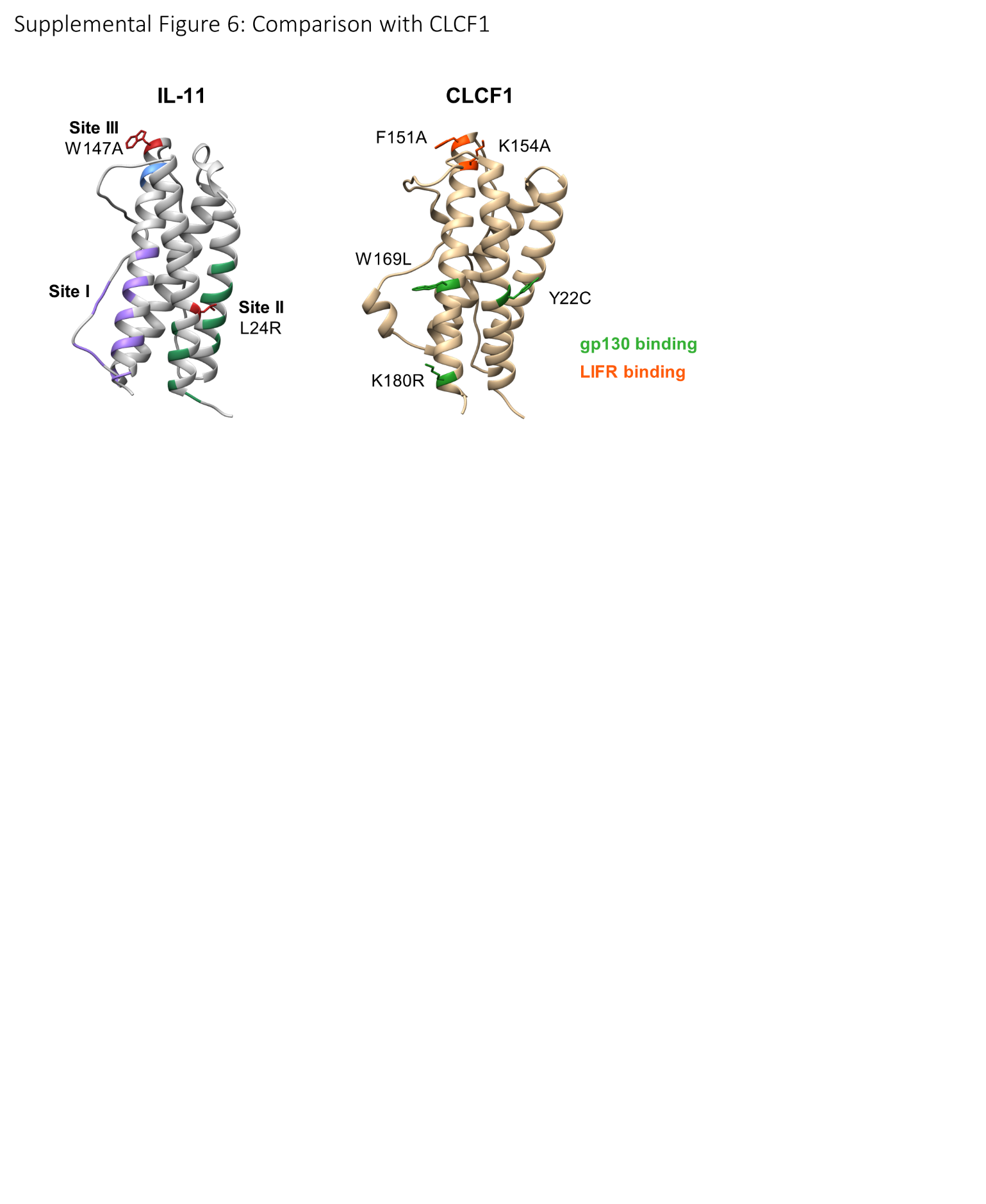


**Figure S8: Comparison of IL-11 and related IL-6 family member CLCF1**. Crystal structure (PDB: 6O4O) of IL-11 with purported Site I (IL-11R binding residues) in purple and Site II and Site III (gp130 binding residues) in green and blue respectively. L24R and W147A are shown in red. Alpha fold predicted structure of CLCF1 with mutations found to disrupt gp130 binding shown in green and mutations found to disrupt LIFR binding shown in orange.^8,54^
